# Supplementary material for: Impact of Preoperative CT-Measured Sarcopenia on Clinical, Pathological, and Oncological Outcomes After Elective Rectal Cancer Surgery
Source: Diagnostics (Basel). 2025 Mar 5;15(5):629. doi: 10.3390/diagnostics15050629 (PMC11899399; doi:10.3390/diagnostics15050629)
Supplement: Supplementary file 1 [file diagnostics-15-00629-s001.zip › diagnostics-3470922-supplementary.pdf]

Supplementary material, Table S1. Cox proportional hazard regression analysis of factors influencing distant recurrence

	Univariate Analysis		Multivariate Analysis	
	**HR (95% CI)**	***P*-Value***	**HR (95% CI)**	***P*-Value***
**Age**	1.008 (0.984-1.034)	0.512		
**BMI**	1.004 (0.941-1.071)	0.901		
**ASA ≥ 3**	1.373 (0.711-2.651)	0.345		
**Sarcopenia**	3.375 (1.489-7.652)	**0.004**	2.989 (1.309-6.826)	**0.009**
**SMRA**	0.956 (0.923-0.991)	**0.013**	0.970 (0.934-1.008)	0.119
**IMATI**	1.049 (0.979-1.124)	0.175		
**CRM involvement**	0.665 (0.160-2.774)	0.576		
**Neoadjuvant treatment**	1.470 (0.732-2.953)	0.279		
**Postoperative complications**	1.780 (0.949-3.339)	0.072	1.538 (0.815-2.902)	0.184
**T3/T4 status**	2.585 (1.225-5.455)	**0.013**	2.043 (0.926-4.504)	0.077
**N1/2 status**	1.695 (0.904-3.179)	0.100	1.383 (0.714-2.680)	0.336
**R1/R2 resection**	1.426 (0.628-3.237)	0.396		

* Significant *p* values (< 0.05) are displayed in bold characters. Variables with a *p* value ≤ 0.1 were candidates for multivariable analysis. HR: hazard ratio; BMI: body mass index; ASA: American Society of Anesthesiologists; SMRA: skeletal muscle radiation attenuation; IMATI: intermuscular adipose tissue index; CRM: circumferential resection margin.

Supplementary material, Table S2. Cox proportional hazard regression analysis of factors influencing local recurrence

	Univariate Analysis		Multivariate Analysis	
	**HR (95% CI)**	***P*-Value***	**HR (95% CI)**	***P*-Value***
**Age**	1.024 (0.975-1.076)	0.340		
**BMI**	1.017 (0.902-1.147)	0.785		
**ASA ≥ 3**	6.631 (1.967-22.354)	**0.002**	3.779 (0.786-18.182)	0.097
**Sarcopenia**	2.376 (0.645-8.795)	0.095	1.803 (0.285-11.410)	0.531
**SMRA**	0.931 (0.870-0.996)	**0.037**	0.923 (0.810-1.051)	0.227
**IMATI**	1.170 (1.076-1.273)	**0.010**	1.007 (0.860-1.178)	0.933
**CRM involvement**	2.775 (0.587-13.123)	0.098	0.538 (0.044-6.505)	0.626
**Neoadjuvant treatment**	1.829 (0.495-6.764)	0.365		
**Postoperative complications**	5.407 (1.462-20.011)	**0.011**	4.814 (0.943-24.575)	0.059
**T3/T4 status**	1.907 (0.571-6.366)	0.294		
**N1/2 status**	1.962 (0.631-6.099)	0.244		
**R1/R2 resection**	2.383 (0.640-8.880)	0.096	0.987 (0.111-8.765)	0.991

* Significant *p* values (< 0.05) are displayed in bold characters. Variables with a *p* value ≤ 0.1 were candidates for multivariable analysis. HR: hazard ratio; BMI: body mass index; ASA: American Society of Anesthesiologists; SMRA: skeletal muscle radiation attenuation; IMATI: intermuscular adipose tissue index; CRM: circumferential resection margin.
